# School principals’ social support and teachers’ basic need satisfaction: The mediating role of job demands and job resources

**DOI:** 10.1007/s11218-022-09730-6

**Published:** 2022-10-31

**Authors:** Jasper Maas, Simone Schoch, Urte Scholz, Pamela Rackow, Julia Schüler, Mirko Wegner, Roger Keller

**Affiliations:** 1 Leiden University, Leiden Institute of Education and Child Studies, Wassenaarseweg 52, 2333 AK Leiden, Netherlands; 2 Zurich University of Teacher Education, Centre for Inclusion and Health in Schools, Lagerstrasse 2, 8090 Zürich, Switzerland; 3 Department of Psychology, Applied Social and Health Psychology, University of Zurich, Binzmühlestrasse 14 / Box 14, 8050 Zürich, Switzerland; 4 Faculty of Natural Sciences, University of Stirling, FK9 4LA Psychology, Stirling, UK; 5 Department of Sports Science, Sport Psychology, University of Konstanz, Universitätsstrasse 10, 78464 Konstanz, Germany; 6 Institute of Human Movement Science, Health Sciences, University of Hamburg, Mollerstrasse 10, 20148 Hamburg, Germany

**Keywords:** Social support, Basic need satisfaction, School principals, Job demands, Job resources

## Abstract

Many teachers report high levels of occupational stress. Teachers’ basic need satisfaction is essential for teachers’ well-being at work. Social support from school principals is assumed to play an important role for teachers’ basic need satisfaction. However, the underlying mechanisms of the relationship between social support from school principals and teachers’ basic need satisfaction are mostly unknown. Previous research suggests that job demands and job resources may play an important mediating role. Therefore, we examine whether teachers’ perceived job demands and job resources serve as mediators between social support from the school principal and teachers’ basic need satisfaction. Using longitudinal data of *N* = 1071 teachers over the course of one school year, we applied structural equation modelling to test the hypothesised mediation model. Results showed that the relationship between social support from the school principal and teachers’ basic need satisfaction was mediated by teachers’ perceived job demands and job resources. Particularly, the job demand ‘unclear organisational conditions’ and job resource ‘social support from colleagues’ indicated the strongest effects on teachers’ basic need satisfaction. These findings emphasise the responsibility of school principals to provide social support to their teachers and create a well-structured and supportive workplace. In doing so, school principals contribute to a work environment in which teachers can thrive.

## Introduction

Many teachers see themselves as being subjected to increasing requirements: In Switzerland for example, 44% reported to have experienced situations in which the pressures were almost unbearable (Kunz Heim et al., 2014). Moreover, studies show that a significant number of teachers experienced impaired well-being during their work such as emotional exhaustion (e.g., Bauer 2009; Hakanen et al., 2006; Skaalvik & Skaalvik, 2011). According to the self-determination theory (SDT; Deci & Ryan 2000) the satisfaction of the basic psychological needs for autonomy, competence, and relatedness support employees’ well-being (Van den Broeck et al., 2016). The satisfaction of employees’ basic needs is an important leadership task (Gagné & Deci, 2005; Van den Broeck et al., 2008). Thereby, supportive school principals were found to be beneficial for teachers’ basic need satisfaction (BNS, Collie et al., 2016; Klassen et al., 2012). However, it is not clear which underlying mechanisms explain the association between social support from the school principal and teachers’ BNS.

## Theoretical framework

### School principals’ social support and basic need satisfaction

Social support is one of the most important aspects of social interactions (Knoll & Kienle, 2007) and refers to the qualitative aspect of helping between two parties. Several types of these supportive interactions can be distinguished. *Perceived* or anticipated social support involves the support that a person expects to be available in his/her social network when help is needed (Knoll & Kienle, 2007). Studies indicated that perceived social support is more a stable rather than a modifiable characteristic (e.g., Sarason et al., 1987). Thus, perceived social support is somewhat independent from the actual behaviour of a specific network member and therefore not an optimal characteristic to measure supportive interactions (Knoll & Kienle, 2007). In contrast, *received* social support is a retrospective report that reflects actual support transactions from specific network members (Uchino, 2009). The present study focusses on received social support.

The self-determination theory (Deci & Ryan, 2000) suggests that well-being is significantly influenced by the satisfaction of the three universal, innate basic psychological needs for autonomy, competence, and relatedness. The need for autonomy refers to the feeling of being in control of one’s decision, being the origin of one’s choices, a sense of ownership, and not being controlled by external forces (Deci & Ryan, 2000). The need for competence is defined as the feeling of mastery and efficacy in accomplishing goals and acquiring new skills (Deci & Ryan, 2000). The need for relatedness involves a feeling of respect, understanding, connectedness, and being significant to others (Deci & Ryan, 2000). BNS is widely known to contribute to employees’ well-being (Van den Broeck et al., 2016).

The provision of social support by leaders is a central aspect in satisfying the basic needs of employees (Gagné & Deci, 2005; Van den Broeck et al., 2008). The receipt of social support is assumed to generate an energising and motivational process by satisfying basic psychological needs (Bakker et al., 2014; Gagné & Deci, 2005). Comparably, empirical research demonstrated that autonomy support from school principals was beneficially related to teachers’ BNS (Collie et al., 2016; Klassen et al., 2012). Although this indicates that there is already empirical evidence for the relationship between social support from the school principal and teachers’ BNS, the concrete mechanism is still unclear. We suggest that this mechanism can be found in the characteristics of the concrete work situation.

### School principals’ influence on teachers’ job demands and job resources

According to the Job Demands-Resources model (JD-R model) the concrete work environment can be classified in job demands and job resources (Bakker & Demerouti, 2017). Job demands are job characteristics that require long-term physical or mental effort and are therefore associated with physical and/or mental costs (Demerouti et al., 2001). Job resources are functional in achieving work goals, reduce physical and/or mental costs, and stimulate personal growth and development (Demerouti et al., 2001). Research suggests leaders’ behaviour to be an antecedent of job demands and job resources (Bakker & Demerouti, 2017; Schaufeli, 2015; Vincent-Höper & Stein, 2019). We focus on the receipt of social support from the leader as a central element of leadership behaviour. The instrumental function of social support (Schwarzer & Knoll, 2007) is manifested by the process in which leaders shape and adjust job demands and job resources (cf. Schaufeli, 2015; Vincent-Höper & Stein, 2019). Such adjustments are defined as job redesign which involve top-down changes in specific aspects of the job, tasks or roles of the individual employee (Bakker & Demerouti, 2014). For example, school principals can allocate the task of organising extracurricular activities or assist in demanding tasks such as a difficult parent conversation. Moreover, the receipt of social support from the leader has an emotional coping function (Schwarzer & Knoll, 2007). This covers aspects such as encouragement, respect, and comfort which can support teachers in coping with job demands and let them perceive their work more positive. Furthermore, the receipt of social support from the school principals can also serve as exemplary behaviour for the teaching team. By behaving in a way that sets a positive example for employees, leaders can create a respectful, constructive, and supportive work environment in which helping behaviour among colleagues can flourish (Bass & Riggio, 2006; Nielsen & Daniels, 2011). Therefore, the receipt of social support from the school principal may result in more supportive interactions among school team members. In sum, the influence of school principals on teachers’ job demands and job resources may explain the beneficial association between social support from the school principal and teachers’ BNS.

### Job demands, job resources and basic need satisfaction

In the present study, we focus on the mediating role of the two job demands ‘perceived time pressure’ and ‘unclear organisational conditions’ and on the job resource ‘received social support from colleagues’. A recent large-scale study among teachers showed that time pressure and unclear organisational conditions stand out as important sources of strain (Brägger, 2019). Social support, in turn, was found to be a central part of a good working climate in schools (Rothland, 2013).

The physical and mental costs associated with job demands thwart teachers’ satisfaction of their needs for autonomy, competence, and relatedness, whereas the energising and motivational function of job resources contribute to teachers’ BNS (Bartholomew et al., 2014; Fernet et al., 2013). Thus, perceived time pressure and unclear organisational conditions may frustrate teachers’ BNS due to the mental efforts that are required to cope with these job demands. Such an effort might be to cope with external control that can increase due to these demands. Therefore, teachers’ leeway to make autonomous decisions and their feeling of ownership may be limited (cf. Bartholomew et al., 2014), hence frustrating the satisfaction of the need for autonomy. Comparable, time pressure and unclear organisational conditions are assumed to frustrate the satisfaction of the need for relatedness: Teachers may have less time to interact and cooperate with colleagues due to time pressure, and because of unclear organisational conditions teachers might turn away from the organisation and colleagues and isolate themselves. Yet, satisfaction of the need for competence might be differently affected by time pressure and unclear organisational conditions. A clear and well-structured context is assumed to be critical for achieving goals and thus for the experience of competence.

Whereas unclear organisational conditions might thwart feelings of competence because they complicate accomplishing work tasks since goals and tasks are unclear, time pressure might result in stronger feelings of mastery, efficacy, and professional competence because accomplishing work tasks within limited time can be experienced as a personal achievement (cf. Crawford et al., 2010). There is empirical evidence to expect that time pressure can have both detrimental and beneficial effects on employees (Widmer et al., 2012). Among teachers time pressure has been shown to be positively associated with both burnout symptoms and job satisfaction (Skaalvik & Skaalvik, 2017).

Contrary, the job resource ‘received social support from colleagues’ might have an energising and motivational effect by promoting teachers’ BNS. Interpersonal relationships at the workplace are important for employees’ BNS (Manganelli et al., 2018). Moreover, social support from colleagues was previously found to contribute to teachers’ needs for autonomy, competence, and relatedness (Doménech-Betoret et al., 2015; Fernet et al., 2013). Consequently, we argue that received social support from colleagues satisfies teachers’ basic needs for autonomy, competence, and relatedness.

### The present study

**Fig. 1:**
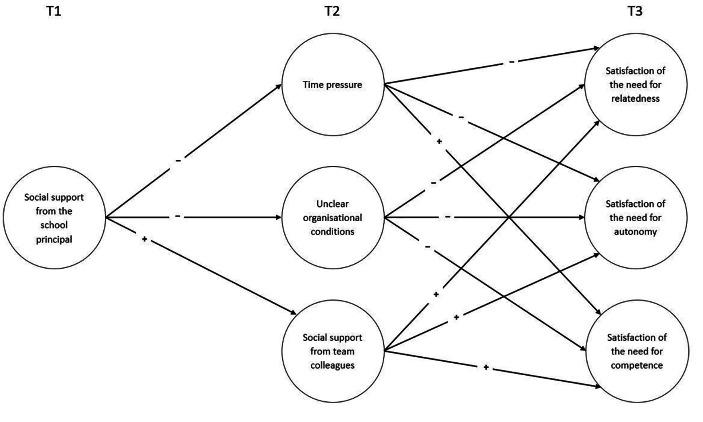
Conceptual research model

In summary, we focus on the mediating role of perceived time pressure, unclear organisational conditions, and received social support from colleagues in the relationship between received social support from the school principal and teachers’ satisfaction of the needs for autonomy, competence, and relatedness (see Fig. 1). We hypothesise:

#### Hypothesis 1

Received social support from the school principal is positively related to teachers’ satisfaction of the basic need for autonomy, competence, and relatedness (direct effect).

#### Hypothesis 2

The positive association between received social support from the school principal and teachers’ satisfaction of the basic needs for autonomy, competence, and relatedness is mediated by the job demands ‘time pressure’ and ‘unclear organisational conditions’ and the job resource ‘received social support from colleagues’ (mediated effects).

#### Hypothesis

*a*: Time pressure is negatively related to the satisfaction of the needs for autonomy and relatedness and positively related to the satisfaction of the need for competence.

#### Hypothesis

*b*: Unclear organisational conditions are negatively related to the satisfaction of the needs for autonomy, competence, and relatedness.

#### Hypothesis

*c*: Received social support from colleagues is positively related to the satisfaction of the basic needs for autonomy, competence, and relatedness.

## Methods

### Procedure

The hypotheses were tested in a sample of teachers at primary (pupils aged 5 to 12 years) and lower-secondary compulsory school level (pupils aged 13 to 15 years) in the German-speaking part of Switzerland. The participants were recruited through teacher organisations. The participants registered individually for participation by giving written informed consent. Eligible for participation were teachers that: teach at primary or lower secondary school level, have a minimum workload of 10 lessons per week, and work at a school with a formal school principal^1^. The participants were requested to fill out online questionnaires in the school year 2017/2018. The first questionnaire was assessed in September 2017 (T1), the second in January 2018 (T2), and the third May 2018 (T3). The completion of each questionnaire took 45 min on average. As compensation for their participation the participants received a voucher worth 25 Swiss francs for each of the three completed questionnaires.

### Participants

In total, *N* = 1365 teachers gave written informed consent. Of these, *n* = 1082 (79.3%) completed all three questionnaires. Over the course of the three measurement points *n* = 283 participants dropped out (20.7%). Dropout was unrelated to the control variables and to the model variables. From the total sample of *N* = 1365 participants, data of *n* = 294 participants were excluded: *n* = 110 participants did not meet the conditions for participation, *n* = 154 participants were special education teacher and worked with very small groups of students in contrast to the other study participants working groups of around 20 students, *n* = 28 participants did not have the same school principal during the entire period of data collection (T1 – T3), and *n* = 2 participants gave implausible answers. The final sample consisted of *N* = 1071 teachers (79.5% females, 18.3% males, 2.1% persons did not report gender). Age ranged between 22 and 65 years (*M* = 42.8 years, *SD* = 11.27). School level was distributed as follows: 74.7% teachers at primary school level, 23.1% teachers at lower secondary school level, and 2.2% teachers at both primary and secondary school level. The mean teaching experience was *M* = 17.3 years (*SD* = 10.86), and the mean workload was *M* = 80.8% of a full-time equivalent (*SD* = 20.17). Although the study did not aim to obtain representative data, these sample characteristics corresponded largely to the population of teachers in the German-speaking part of Switzerland in the schoolyear 2016/17 (Federal Statistical Office, 2018).

### Measures

*Received social support from the school principal* was assessed at T1 using an adapted version of the Berlin Social Support Scales (Schulz & Schwarzer, 2003). The original scale was reformulated to fit the school setting. The instrument consisted of the subscales emotional and instrumental support which were, due to a strong correlation (*r* = .85), merged to create one scale. Three negatively worded items were excluded because they did not load on the intended factor but constituted a separate factor. This is a phenomenon that appears to be rather common for negatively worded items (Barnette, 2000). This resulted in a scale consisting of 10 items with a 6-point response format (anchors of *not true at all* and *absolutely true*). A sample item was ‘My principal assured me that I can completely rely on him/her’. Cronbach’s alpha was 0.94 (*M* = 4.11, *SD* = 1.26).

*Basic need satisfaction* was assessed at T3 with the German version of the Work-related Basic Need Satisfaction scale (Van den Broeck et al., 2010). The instrument consisted of the three subscales *autonomy*, *competence*, and *relatedness*, with each six items. A sample item for autonomy was ‘I feel free to do my job the way I think it could best be done’, for competence ‘I feel competent in my job’, and relatedness ‘At work I feel as part of a group’. Participants rated these items on a 5-point scale (anchors *does not apply at all* and *applies completely)*. Cronbach’s alpha was 0.82 for satisfaction of the need for autonomy (*M* = 3.66, *SD* = 0.71), Cronbach’s alpha was 0.79 for the need for competence (*M* = 4.21, *SD* = 0.53), and Cronbach’s alpha was 0.84 for the need for relatedness (*M* = 3.96, *SD* = 0.78).

*Perceived time pressure* was assessed at T2 using the subscale ‘time pressure’ of the questionnaire on psychological strain among teachers in Germany (Nübling et al., 2008). The scale consisted of three items with a 5-point response format (anchors *does not apply at all* and *applies completely*). A sample item was ‘I was frequently under time pressure’. Cronbach’s alpha was 0.74 (*M* = 3.04, *SD* = 0.93).

*Unclear organisational conditions* were assessed at T2 and consisted of four items from the instrument S-Tool in Schools (Krause & Böschenstein, 2014). These items express school specific demands: I felt burdened by… ‘unclear regulations regarding core business and additional tasks of the school’, ‘changes in the school organisation’, ‘school reforms’, and ‘unclear regulations regarding responsibilities and competencies’. Participants rated these items on a 5-point scale (anchors *does not apply at all* and *applies completely)*. Cronbach’s alpha was 0.74 (*M* = 2.74, *SD* = 0.98) and factor loadings ranged from 0.49 to 0.74. This can be considered to be sufficient to treat the four items as one-dimensional (Bortz & Schuster, 2010).

*Received social support from colleagues* was assessed at T2 with an adapted version of the Berlin Social Support Scales (Schulz & Schwarzer, 2003). In line with the scale *received social support from school principal*, the original scale was reformulated to fit the school setting and three negatively worded items were excluded. The final scale therefore consisted of 10 items with a 6-point response format (anchors of *not true at all* and *absolutely true*). A sample items was ‘My team colleagues assured me that I can rely completely on them’. Because the two subscales emotional and instrumental social support correlated rather high (*r* = .60) and to correspond to ‘received social support from the school principal’, the subscales emotional and instrumental support were merged into one scale. Cronbach’s alpha was 0.94 (*M =* 4.66, *SD* = 1.08).

### Data-analytical strategy

The hypothesised model was tested with structural equation modelling using R version 3.4.3 (R Core Team, 2017) and the Lavaan package version 0.6-3 (Rosseel, 2012). To estimate the hypothesised model, we used robust maximum likelihood estimation (MLR), which is robust against violations of normality assumptions (Lai, 2018). Full information maximum likelihood estimation (FIML) was used to handle missing data (Graham & Coffman, 2012). First the measurement model was tested with confirmatory factor analysis followed by testing the hypothesised full mediation model and comparing it with a partial mediation model.

Research showed that job tenure is an important predictor for teachers to experience stress in a way that years of employment are negatively related to the experience of stress (Bradley, 2007). This assumption was also indicated by zero-order correlations between number of years of employment as a teacher and received social support from the school principal and from colleagues, unclear organisational conditions, and satisfaction of the need for competence^2^. Therefore, we included the total number of years of employment as a teacher as control variable in the hypothesised model^3^.

To determine model fit we used four fit indices: Chi-square/*df* ratio (χ²/df), the Root Mean Square of Approximation (RMSEA), Standardised Root Mean Residual (SRMR), and the Comparative Fit Index (CFI). χ²/df ≤ 5 indicates good model fit (West et al., 2012). RMSEA ≤ 0.06 indicates a good fit, SRMR ≤ 0.08 is considered as a good fit, and the incremental fit indices CFI > 0.95 reflects good fit (Hu & Bentler, 1999).

For testing the proposed mediated effects, we used bias-corrected bootstrapping. This involved computing bias-corrected 95% confidence intervals from 1000 resamples (MacKinnon et al., 2004; Preacher & Hayes, 2008).

## Results

### Preliminary analyses

Descriptive statistics and zero-order correlations between the model variables are shown in Table 1. In support of hypothesis 1 received social support from the school principal and teachers’ satisfaction of the needs for autonomy, competence, and relatedness were significant positively related to each other and ranged from *r* = .10 to *r* = .27 (*p* < .01). Moreover, perceived time pressure, unclear organisational conditions, and social support from colleagues were in the expected direction significant related to social support from the school principal and teachers’ satisfaction of the needs for autonomy, competence, and relatedness, respectively.

**Table 1:** Zero-order correlations between model variables (*N* = 1071)

Variable	1	2	3	4	5	6
1. Social support from the school principal (T1)						
2. Perceived time pressure (T2)	-.09*					
3. Unclear organisational conditions (T2)	-.31***	.33***				
4. Social support from colleagues (T2)	.26***	-.02	-.16***			
5. Satisfaction of the need for relatedness (T3)	.27***	-.10**	-.20***	.52***		
6. Satisfaction of the need for autonomy (T3)	.27***	-.33***	-.41***	.24***	.36***	
7. Satisfaction of the need for competence (T3)	.10**	-.16***	-.13***	.10**	.26***	.44***

*Note*. T1 first measurement point, T2 second measurement point, T3 third measurement point. *p < .05. **p < .01. ***p < .001.

### Main analyses

To test the hypothesised mediation model (see Fig. 1) we first estimated the measurement model. The initial measurement model yielded acceptable fit to the data (χ²/df = 3.19, RMSEA = 0.048, SRMR = 0.052, CFI = 0.902). Inspection of the modification indices showed that covariances between the error terms of unclear organisational conditions would significantly improve the model^4^ (Satorra-Bentler scaled ∆χ² (2) = 272.70, *p* < .001). This resulted in a measurement model with sufficient model fit (see Table 2).

**Table 2:** Fit indices of measurement model, structural model, and model comparison (*N* = 1071)

Model	χ² (df)	χ²/df	RMSEA	SRMR	CFI	TLI	Satorra-Bentler scaled ∆χ²
1. Measurement model	2777.65 (960)	2.89	.044	.045	.913	.906	
2. Full mediation model	2868.87 (967)	2.97	.046	.052	.909	.902	
3. Partial mediation model	2862.86 (964)	2.97	.046	.052	.909	.902	1 vs. 2 ∆χ² (3) = 6.66

*Note.* The partial mediation model includes direct effects from received social support from the school principal on the satisfaction of all three basic needs. All models are estimated with the robust maximum likelihood estimator; RMSEA = root mean square error of approximation; SRMR = standardised root mean square residual; CFI = comparative fit index; TLI = Tucker–Lewis index.

The hypothesised full mediation model reflected sufficient fit to the data and the partial mediation model did not have a significantly better or impaired model fit (see Table 2). Thus, including direct effects from received social support from the school principal at T1 on teachers’ BNS at T3 did not represent the observed data better than the full mediation model. Because the full mediation model was the most parsimonious this model was preferred (West et al., 2012).

#### Received social support from the school principal and teachers’ BNS

**Fig. 2:**
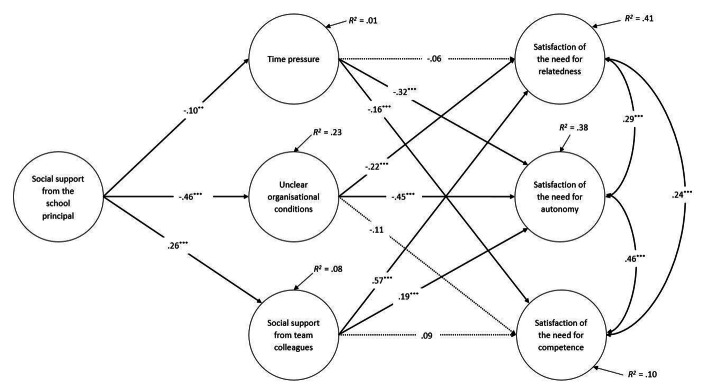
Full mediation model, standardised regression coefficients and explained variance, controlled for job tenure. * *p* < .05. ** *p* < .01. *** *p* < .001. R^2^ = explained variance. Dotted lines represent insignificant path

In support of hypothesis 2 bootstrap analysis showed that using the full mediation model, effects of received social support from the school principal on teachers’ BNS were mediated by the proposed job demands and job resource (see Table 3). The total indirect effects on the satisfaction of the needs for autonomy and relatedness were large (satisfaction of the need for autonomy: β = 0.30, p < .001; satisfaction of the need for relatedness: β = 0.26, *p* < .001), while the total indirect effect on the need for competence was small (β = 0.10, *p* < .001). These effects are also reflected by the explained variance of the needs for autonomy (38%), competence (10%), and relatedness (41%) (see Fig. 2).

**Table 3:** Bootstrapped standardised regression coefficients of indirect and total effects of social support from the school principal on teachers’ satisfaction of the needs for autonomy, competence, and relatedness via time pressure, unclear organizational conditions, and social support from colleagues

Independent variable	Criterion variable	Total indirect effect	Specific indirect effect		
			Perceived time pressure	Unclear organisational conditions	Received social support from colleagues
Received social support from the school principal	Autonomy	.30***	.04**	.21***	.05***
	Competence	.10***	.02*	.05*	.03
	Relatedness	.26***	.01	.10***	.16***

*Note.* *p < .05. **p < .01. ***p < .001

### Job demands and job resources as mediators

The full mediation model shows that perceived time pressure, unclear organisational conditions and social support from colleagues mediated the association between received social support from the school principal and teachers’ BNS (see Table 3; Fig. 2). Yet, the mediating job demands and job resource differ in their specific indirect effects on teachers’ BNS. Perceived time pressure mediated the effects of received social support from the school principal on the satisfaction of the needs for autonomy (β = 0.04, *p* < .01) and competence (β = 0.02, *p* < .05), while it did not mediate the effect on the satisfaction of the need for relatedness (β = 0.01, *p* > .05). Unclear organisational conditions, in turn, mediated the effects of social support from the school principal on the satisfaction of all three basic needs: The indirect effect on the need for autonomy was β = 0.21 (*p* < .001), on the need for competence was β = 0.05 (*p* < .05), and on the need for relatedness was β = 0.10 (*p* < .001). The job resource social support from colleagues mediated the effect of social support from the school principal on the satisfaction of the need for relatedness (β = 0.16, *p* < .001), and on the need for autonomy (β = 0.05, *p* < .001). The mediated effect on the satisfaction of the need for competence through social support from colleagues was not significant (β = 0.03, *p* > .05).

Besides the indirect effects, the examined job demands and job resource had differential direct effects on teachers’ BNS (see Fig. 2). The job demands ‘perceived time pressure’ and ‘unclear organisational conditions’ were negatively related to the satisfaction of the need for autonomy. Regarding the satisfaction of the need for competence, ‘perceived time pressure’ was the only job demand which was significantly negatively related to satisfaction of the need for competence. With regard to the satisfaction of the need for relatedness only the job demand ‘unclear organisational conditions’ was negatively related to the satisfaction of the need for relatedness. Thus, hypotheses 2a and 2b are only partly supported by our results.

With regard to the job resource ‘received social support from colleagues’ results showed that it contributed to the satisfaction of the need for relatedness and the satisfaction of the need for autonomy. The satisfaction of the need for competence was unaffected by received social support from colleagues. Therefore, hypothesis 2c was only partly confirmed.

## Discussion

The present study aimed to test whether the positive effect from received social support from the school principal on teachers’ BNS is mediated by teachers’ perception of the job demands time pressure, unclear organisational conditions, and the job resource social support from colleagues. Results showed that the benefit of received social support from the school principal for teachers’ satisfaction of the needs for autonomy, competence, and relatedness can be explained by perceived time pressure, unclear organisational conditions, and received social support from colleagues. This supports previous research that showed that leadership behaviour is an antecedent of job demands and job resources (Schaufeli, 2015; Vincent-Höper & Stein, 2019). Moreover, it highlights the responsibility of school principals to shape a work environment in which teachers can thrive. The results also suggest that teachers’ basic needs are not satisfied merely because of receiving social support from the school principal, but the social support that school principals provide influences job demands and job resources and, therefore, enhances teachers’ BNS. It is important to note that, although school principals’ leeway to change job demands and job resources might be limited due to imposed legal and school administration regulations, adjustments in the work of individual teachers may still be possible. Shaping and adjusting work tasks, responsibilities, and competencies of the individual employee, defined as job redesign (Bakker & Demerouti, 2014), can be perceived by teachers as instrumental social support from their school principal.

The positive effect of received social support from the school principal on teachers’ BNS is in line with research that emphasised the importance of supportive school principals for the satisfaction of teachers’ basic needs (Collie et al., 2016; Klassen et al., 2012). Our results point out that especially the needs for autonomy and relatedness are beneficially related to received social support from the school principal. However, the need for competence seems to be only little affected by social support from the school principal. This also applies to received social support from colleagues. Previous studies also reported that social support at the workplace seems to contribute primarily to the needs for autonomy and relatedness, while it hardly affects the need for competence (Fernet et al., 2013; Van den Broeck et al., 2010). The needs for autonomy and relatedness reflect rather social needs that can only be satisfied in relation to others (Ryan & Solky, 1996). The need for competence, however, seems more independent of the quality of social relations. Theoretically, feelings of competence could arise without social relations.

Our results showed that perceived time pressure is harmfully related to the satisfaction of the needs for autonomy and competence, while the need for relatedness stays unaffected. It seems that teachers still feel related to their colleagues regardless of the time pressure they perceive. This contradicts hypothesis 2a as we expected time pressure to be harmfully related to the satisfaction of the needs for autonomy and relatedness, but beneficially related to the satisfaction of the need for competence. Whether teachers perceive time pressure as a hindrance that costs effort, or as an energising challenge that promotes motivation, might depend on the extent to which teachers experience loss of control while being under time pressure (Kühnel et al., 2012).

Unclear organisational conditions are negatively related to teachers’ satisfaction of the needs for autonomy and relatedness, whereas it does not seem to affect the need for competence. This partly supports hypothesis 2b as we expected negative relationships with the satisfaction of all three basic needs. The association with the need for autonomy is quite strong which suggests that unclear regulations, school reforms, and implementation of a new curriculum can reduce feelings of being in control of one’s decision and a sense of ownership. One reason for the fact that the need for competence was unrelated to unclear organisational conditions might be, that feelings of mastery and efficacy in accomplishing goals and acquiring new skills might depend more on the core task of teaching students in the classroom than on organisational conditions.

### Strengths and limitations

Taken together, the study has several strengths. First, we were able to contribute to a more detailed understanding of how school principals might positively affect teachers’ BNS, by examining mediating mechanisms. To the best of our knowledge, this has not been done before. Second, the longitudinal design with three measurement points over the course of one school year enables to gain a detailed insight into teachers’ experiences of their work across one school year. Third, although three measurement points meant time consuming study participation for the participants, we had a very low dropout rate and a large sample size of over thousand teachers. As far as we know, this is one of the biggest teacher samples in the German speaking part of Switzerland.

Yet, there are also some limitations that need to be mentioned. First, despite the longitudinal design, we cannot draw conclusions on causality. Influence of a third variable on the relationships found in the present study cannot be ruled out (Mackinnon & Pirlott, 2015). Only studies using an experimental design enable to unfold causal relationships. Such designs would also assist in examining potential reciprocity of the relationships between social support, job demands, job resources, and BNS. Yet, there are ethical limitations for these kinds of studies when it comes to the job demands, so correlational designs are in parts the only designs available for some of our research questions.

Second, we used only self-reports and therefore it could be argued that common method variance (CMV) affected our results (Podsakoff et al., 2003). It is, however, also argued that CMV does not pose a major problem and is overstated (Spector, 2006). We chose to use self-reports because we were interested in the personal experience of teachers. Reports by others would not be accurate enough and the use of more ‘objective’ indicators also has shortcomings, such as observers’ bias, halo and stereotype effects (Kerlinger & Lee, 2000).

## Conclusion

Received social support from the school principal is an important factor that significantly contributes to the satisfaction of teachers’ BNS. Our results show that this relationship is mediated by job demands (i.e., time pressure and unclear organisational conditions), and job resources (i.e., social support from colleagues). Whereas time pressure and unclear organisational conditions represent important risk factors for satisfying the need for autonomy, received social support from colleagues significantly contributes to the need for relatedness. This emphasises the importance of well-structured, clear, and predictable regulations, of autonomy during organisational changes, and the value of supportive colleagues. Accordingly, the social support from school principals can support teachers’ BNS in three ways: (1) By organising, regulating, and allocating work tasks, responsibilities, and competencies. (2) By supporting teachers in coping with job demands and encourage them to perceive work more positive. (3) By showing exemplary behaviour that creates a supportive atmosphere. Doing so, school principals can reduce job demands and promote job resources which creates opportunities for teachers’ basic psychological needs to be satisfied.

## Data Availability

Data and R syntax are available on the Open Science Framework at https://osf.io/269cs/?view_only=9a93e1bea6be40358f434571c7036a46.

## Footnotes

1 At the time of the data collection not all schools in the German speaking part in Switzerland had an organisational structure which included the formal position of school principal.

2 Received social support from the school principal (*r* = − .12, *p* < .001), received social support from colleagues (*r* = − .13, *p* < .001), unclear organisational conditions (*r* = .16, *p* < .001), and satisfaction of the need for competence (*r* = .18, *p* < .001).

3 Inclusion of this control variable did not result in substantial changes in the direction of effects and effect sizes.

4 Although this post hoc adjustment is generally discouraged because of the risk of chance capitalisation (MacCallum et al., 1992), we included it for substantive reasons: Two items referred to unclear regulations regarding tasks, responsibilities, and competencies, whereas two other items referred to organisational changes in the curriculum and teaching methods. These factors had some error variance in common, but all four items also constituted the overarching construct ‘unclear organisational conditions’.
